# Perceptions of barriers and facilitators to implement programs for prevention of mother-to-child transmission of HIV-Mozambique

**DOI:** 10.1590/1980-220X-REEUSP-2021-0353

**Published:** 2022-02-11

**Authors:** Olimpia Buleza Lamucene, Margarita Bernales, Lisette Irarrázabal Vargas, Lilian Ferrer Lagunas

**Affiliations:** 1 Pontificia Universidad Católica de Chile, Facultad de Medicina, Escuela de Enfermería, Santiago, Chile.

**Keywords:** HIV, Women, Pregnant Women, Nursing, HIV, Mulheres, Gestantes, Enfermagem, VIH, Mujeres, Mujeres Embarazadas, Enfermería

## Abstract

**Objective:**

To understand the perspectives of pregnant and postpartum women living with HIV in Sofala, Mozambique, regarding barriers and facilitators to following Prevention of Mother-to-Child Transmission (PMTCT) recommendations.

**Method:**

Qualitative study conducted in three health centers and with a peer support group of women living with HIV, between October 2020 and March 2021. We applied purposeful sampling, semi-structured interviews, and content analysis.

**Results:**

Among the barriers that emerged were the social stigma associated with HIV-positive status and fear of discrimination, side effects of medications, economic barriers, and denial of diagnosis/treatment. As facilitating factors: peer support networks and inspiration, innate concern for health and family. Finally, they recommend that the community should become more educated about HIV.

**Conclusion:**

The results of this study give a broad understanding of the experience of women living with HIV in this province, making it possible to focus strategies in improving the care of women in PMTCT programs in Mozambique.

## RESUMEN

**Objetivo:**

Comprender las perspectivas de las mujeres embarazadas o en posparto viviendo con VIH en Sofala, Mozambique, respecto a las barreras y facilitadores para seguir las recomendaciones para la Prevención de Transmisión Materno Infantil (PTMI).

**Método:**

estudio cualitativo realizado en tres centros de salud y con un grupo de apoyo entre pares de mujeres viviendo con VIH, entre octubre de 2020 y marzo de 2021. Aplicamos muestreo intencional, entrevistas semiestructuradas y análisis de contenido.

**Resultados:**

Surgieron como barreras el estigma social que conlleva la condición de ser VIH positivo y el miedo a la discriminación, los efectos secundarios de los medicamentos, barreras económicas y la negación al diagnóstico/tratamiento. Como factores facilitadores: redes de apoyo e inspiración de pares, innata preocupación por la salud y la familia. Finalmente, ellas recomiendan que la comunidad se eduque más en materia de VIH.

**Conclusión:**

Los resultados de este estudio dan una comprensión amplia acerca de la experiencia de mujeres viviendo con VIH en esta provincia, posibilitando focalizar estrategias para mejorar la atención de las mujeres en los programas de PTMI en Mozambique.

## RESUMO

**Objetivo:**

Compreender as perspectivas das mulheres grávidas ou puérperas vivendo com HIV em Sofala, Moçambique, sobre as barreiras e facilidades para seguir as recomendações para a Prevenção da Transmissão Materno-Infantil (PMTCT).

**Método:**

estudo qualitativo realizado em três centros de saúde e com um grupo de apoio de pares de mulheres vivendo com HIV, entre outubro de 2020 e março de 2021. Aplicou-se amostra intencional, entrevistas semiestruturadas e análise de conteúdo.

**Resultados:**

O estigma social associado a ser HIV positivo e o medo da discriminação, efeitos colaterais dos medicamentos, barreiras econômicas e negação do diagnóstico/tratamento surgiram como barreiras. Como fatores facilitadores: redes de apoio e inspiração dos pares, preocupação inata com a saúde e a família. Finalmente, eles recomendam que a comunidade se eduque mais sobre o HIV.

**Conclusão:**

Os resultados deste estudo proporcionam uma compreensão ampla da experiência das mulheres que vivem com o HIV nesta província, possibilitando centrarse em estratégias para melhorar o atendimento às mulheres nos programas de PTMI em Moçambique.

## INTRODUCTION

Globally 1.8 million children up to 14 years of age were living with the human immunodeficiency virus at the end of 2019^(1)^, with mother-to-child transmission (MTCT) being the main route of transmission^(2)^. In the absence of interventions, MTCT can occur in 15-45% of cases^(2)^. However, effective interventions aimed at minimizing transmission reduce the risk to 5% or less^(2)^. Universal antiretroviral treatment (ART) for all pregnant women, regardless of CD4 defense cell count or clinical stage of infection, known as option B+ has been one of the most recent interventions recommended by the World Health Organization (WHO)^(3)^ to contain MTCT worldwide; this recommendation was adopted by several countries, including the government of Mozambique in 2013^(4)^. In this nation, the latest estimates indicate that 150,000 children under 14 years of age were living with HIV by the end of 2019^(5)^.

The introduction of the Option B+ regimen has overcome barriers of the ART initiation among women, allowing for greater initiation into PMTCT programs^(6)^. However, new challenges arise around retention of these women in the programs. Reports in Mozambique on HIV in pregnant women have reported a PMTCT treatment loss rate of up to 45% within 12 months of initiation^(7)^. These challenges have sparked the interest of countries in understanding the reasons why people living with HIV, including pregnant women, make the decision to start and adhere to HIV treatment and PMTCT programs, as well as why they decide to drop out.

Qualitative studies addressing this issue have identified some barriers and factors that facilitate PMTCT^(8–11)^. A review with both quantitative and qualitative studies from sub- Saharan Africa investigated the reasons for low access, initiation and adherence to ART to prevent MTCT, and the factors found in the qualitative studies are summarized in the *socio-ecological* ^(8)^ model. At the individual level, economic factors, health perception, desire to look healthy, protection of the health of the child, and knowledge about HIV and treatment were the factors that most impacted decision making.

At the community level, barriers included stigma and fear of disclosure, lack of support, and cultural beliefs^(8)^. Barriers at the health system level were poor provider-client relationships, lack of confidentiality^(12)^, and shortages of health personnel^(13)^. In Mozambique, as in other African countries, culture and gender roles influence health-seeking behavior^(11,14)^. Concern for the health of offspring among women may lead them to seek health care during pregnancy, but postpartum abandonment 6 to 12 months later. The risk of transmission during breastfeeding may increase as breastfeeding often lasts for at least 2 years^(9)^.

Based on these findings, it is interesting to investigate the specific barriers and facilitators perceived by women in Sofala, Mozambique. Therefore, a study was conducted to understand the perspectives of pregnant and postpartum women by exploring their barriers and facilitators in following PMTCT recommendations.

## METHOD

### Type of Study

Qualitative study, constructivist paradigm, using a “case study” approach^(15)^. This methodology allows us to know from the perspective and subjectivity of women, how they elaborate the phenomenon of incorporating the treatment into their lives.

### Population

Pregnant and postpartum women living with HIV participated in the study. The inclusion criteria were living with HIV, being older than 18 years, and being or having participated in PMTCT programs. Both the selection of participants and institutions were done by purposeful sampling^(15)^. The sample size was determined by data saturation^(16)^.

### Local

This study was conducted in 3 primary care centers, two urban and one rural. A member of a peer support group of women living with HIV in the province of Sofala in Mozambique also participated.

### Data Collection

Data collection was by means of individual semi-structured interviews, via online, between October 2020 and March 2021, using a question guide previously validated through a pilot interview for the interviews. A midwife at each health center identified potential participants and invited them to the study. The researcher, once in contact with the interested party, explained in detail the purpose and objective of the study and completed the informed consent process orally. She then conducted the video interviews using WhatsApp or Zoom platforms. One person refused to participate due to lack of time. They were conducted in Portuguese, except for two done with local languages (Ndau and Sena). We recruited until we obtained a saturation of information^(16)^. Thirteen interviews were conducted from the health center in a private room and two from the participants’ homes. The interviews lasted between 30 to 70 min. Finally, field notes were taken throughout the data collection process. Each participant received a reimbursement of 200 Mtn (Mozambican currency), equivalent to 3 USD in compensation for the travel expenses of the interviews.

### Data Analysis and Processing

The audio-recorded interviews were transcribed word by word in the language used and then translated into Spanish. To verify the quality of the translations, a person external to the research team, fluent in Portuguese and Spanish, reviewed two randomly selected interviews. Thematic analysis was used to analyze the data.

### Ethical Aspects

This study has the approval of the Ethical-Scientific Committee of Health Sciences of the Pontificia Universidad Católica de Chile, ID: 200814012. In addition, we have the authorization of the directors of the health centers where the participants were recruited, as well as the person in charge of the support group.

## RESULTS

Fifteen women with an average age of 30 years (range: 20-42 years), mostly married with more than 2 pregnancies prior to the interview, participated in the study. All were aware of their HIV status prior to the current pregnancy, including primigravidae. Eight women had been diagnosed at the previous prenatal visit, 8 were in paid employment. The proportion of pregnant women (8) was slightly higher than postpartum.

The analysis of the participants’ narratives identified barriers and facilitators that affect the decision to initiate and continue antiretroviral treatment for PMTCT. Three major themes were identified in the thematic analysis: barriers, facilitators to adhere to the programs, and opportunities for improvement (referring to women’s desires and preferences). These are presented in detail below.

### Barriers that Influence the Initiation, Retention and/or Return to PMTCT Programs

Participants reported having faced or facing challenges related to diagnosis and ART. It was observed: denial of diagnosis and/or treatment, side effects exacerbated by dietary deficits, and finally stigma and fear of discrimination. Other barriers reported to a lesser extent consisted of regimen change dictated by medication and forgetfulness.

### Refusal of Diagnosis and/or Treatment

The denial of the diagnosis experienced by the participants, as well as in what they perceived from other People Living with HIV (PLHIV), is related to the asymptomatic state at the time of diagnosis, lack of trust in health systems, and the belief in other origins of the disease such as witchcraft. This led to late initiation of treatment and/or non-compliance with recommendations.

*I think at first, I didn’t believe. [I was telling myself] I’m fine. So, when I came here, when I tested and I accused [it was positive], at first, I didn’t want to take [the medicine*]. *I didn’t take them properly because I didn’t feel bad and also because, honestly speaking, I didn’t see any reason to take (P4, 31 years old, pregnant)*.

*I know many people who abandoned the treatment because they went to the healer, they were told that they cannot do the treatment, that is, “you are not sick, you are being cheated.” (P10, 42 years old, postpartum).*

Likewise, denial was common among the participants’ partners and led to a lack of support, perceived as a barrier. One woman crying during her story said:

*My husband flatly refused. He told me that, “if you want to move on, the marriage is over, and you will suffer with the children”. And I believed him, leaving the disease, believing in my husband. The baby was born, I breastfed her and the child also got the same disease. (P6, 33 years old, pregnant).*

Thus, the participants’ denial was aggravated by previous prejudices about the disease and by the social environment in which they were inserted.

### Side Effects and Economic Barriers

All the participants in this study reported having some side effects from the drugs, especially at the beginning of treatment. In some cases, it led to temporary discontinuation of treatment. *At first, I was getting dizzy; it was at that point that I said I’d better give up. [I said] if I stop taking it, maybe I won’t feel these things anymore (P13, 25 years old, pregnant).*

The most reported side effects were dizziness, dermatological allergies, hunger, and fatigue, interfering with the participants’ daily activities.

*It was normal for me to take it and the next day be in bed all day. I couldn’t get up or do anything. I couldn’t even take a shower (P8, 29 years old, postpartum).*

The economic barriers mentioned in this study were closely related to side effects. For example, because there were insufficient financial resources to purchase food, taking the medication without food produced greater perception of adverse effects. This participant reported:

*At that time [when I was pregnant] I was starving, I had nothing to eat. Whenever I took the medicine without eating something, I got dizzy, I felt worse; then I started to skip the medication. Now my baby tested positive too.” (P10, 42 years old, postpartum*).

Late initiation and incorrect follow-up of ART was more common among participants who experienced side effects in the absence of symptoms of HIV infection.

*You know, when you are not sick and suddenly you have to start taking a medicine for life and you see that it is hurting you even more, one can get to stop it. For example, I would choose days to take it. When I took today, the next day I didn’t take it. (P4, 31 years old, pregnant).*

Thus, the absence of symptoms at the time of diagnosis, and the scarcity and lack of economic support, aggravated the intolerance to side effects.

### Stigma and Fear of Discrimination

Fourteen of the fifteen interviewees experienced the barrier of stigma, mainly anticipated and perceived stigma. The women feared disclosure of the diagnosis without consent.

*My service partners must not know about it in any way; because if they know someday, oops! My life may end… Because they are these people, I don’t want to lie, who can take me out of being better and make me fall, stress me out until my life is over (P4, 31 years old, pregnant).*

Perceived stigma consisted of the participants’ beliefs of how PLHIV are treated and somehow extrapolate it into to their own cases.

*In the community I can say that there is discrimination because when people realize that you are like that, they move away. They talk about that and that (P8, 29 years old, postpartum).*

Three interviewees reported having suffered discrimination in the form of insults, rejection by neighbors and abandonment by their partners:

*After [the diagnosis] I noticed some difference in my partner. He started to distance himself. First, he ran away, then he didn’t give any explanation as to why he was distant (P2, 27 years old, postpartum)*.

*I have suffered discrimination. A long time ago they gave me soy, beans, and rice [at the hospital]. When I took it home, [the neighbors] saw me. Then they started to say that you can’t eat things from this neighbor here because she has HIV (P13, 25 years old, pregnant).*

As evidenced in the stories, stigma, regardless of its form of expression, had a negative impact, leading the participants to live in fear of the disclosure of their condition.

### Facilitators to Adhere to the Programs

Despite the barriers that many women faced at the time of or immediately after the diagnosis, factors were identified that made it possible to overcome the barriers and motivate the initiation and continuation of treatment. These include concern for health and family well-being, and peer support networks and inspiration.

### Concern for Family Health and Well-Being

All participants, especially among those diagnosed during pregnancy, stated the desire to protect their children from infection as their motivator for pursuing PMTCT.

*My biggest motivation now is my baby; being a mother, I try not to pass on the disease to my child. That’s exactly what makes me want to take [the medications] and always look for [information]. (P4, 31 years old, pregnant).*

Although some participants stated that: *“What drives me, what motivates me the most, besides the pregnancy, is because I love myself, I like myself, I want to look good, healthy and move forward (P1, 28 years old, pregnant),* in most cases this concern for their health is closely related to the desire of taking care of their children and avoid MTCT, as illustrated.

*[My biggest motivation] is to be healthy and to take care of my children. For the children to grow up in good health, not to have something happen to them, and for them to stay healthy. (P5, 25 years old, postpartum).*

*What made me not give up, right? Was for my children. For the sake of my children. Because I started to see, if I don’t take it, I will hurt myself and my children who still need a lot from me as a mother. (P12, 35 years old, pregnant).*

Likewise, feeling healthy as a result of the treatment facilitated its continuation.

*[I continue with the treatment] because I am not very sick anymore, and because now I have nothing to complain about like before. I feel so good in my body. So, I continue with the treatment because I see that it really helps me. (P11, 27 years old, pregnant).*

Thus, the prioritization and concern for the health of the child and the instinct to protect the family and offspring was reinforced by health professionals in their advice during PMTCT consultations.

*They [health professionals], at the time [of starting the prenatal consultation], told me, you can’t stop taking it because you are carrying a life and that life can be born while you are [infected] (P8, 29 years old, postpartum).*

### Peer Support and Inspiration Networks

Emotional support, mainly from spouses, was expressed as a motivator to accept the diagnosis and initiate and/or continue treatment. Among the most valued actions was the acceptance of the participants’ condition and reminders to take the medication.

*[What made me decide not to fail with the medicine] I think it was my husband. He always insisted when the time came, because we decided to take it at the same time. So, when the time came, he would bring the medicine for both of us, give it to me and we would take it together (P4, 31 years old, pregnant).*

All the interviewees reported having a good relationship with their health care providers (HCPs). They reported receiving advice and comfort from them, especially at the time of diagnosis. This encouraged them to follow medical recommendations and overcome some barriers.

*The doctor’s advice gave us a lot of strength, a lot of positive energy, and we started with the treatment. I had no more will to live. It was then that the doctor said no, it is not the end of life, you have to be strong and have the will to live; your life does not end here. I think that if it wasn’t for the advice of people, psychologists, I don’t know what would become of me and my partner (P8, 29 years old, postpartum).*

The support network also extended to the family and community level. Ten participants who had disclosed their diagnosis to family members, other than their spouses, reported receiving their support as incentives to accept and continue treatment.

*I told [about my diagnosis] to my mother and my sister… they support me; they urge me to continue. That time when I had given up, they also encouraged me to come back* (P7, *20 years old, pregnant*).

Finally, peer support or inspiration was common among participants. Inspiration could be from positive outcomes, such as seeing that others in the same situation and treatment looked and felt healthy, but also in the negative outcomes of lack of treatment.

*I lived with someone at home without knowing about her situation; she did not open her mind saying that she was in this situation. She just supported me and said I couldn’t miss a day (P2, 27, postpartum).*

*What has motivated me to follow the treatment is to see that all those who are following the treatment are alive, have a normal health and those who abandon the treatment are disfigured, sick, etc. (P10, 36 years old, postpartum).*

Thus, seeing other people living with HIV healthy because of the treatment or seeing the deterioration in those who did not accept treatment served as motivation to continue treatment.

### Improvement Opportunities

Upon inquiring about the needs and desires of the participants to improve their experience as mothers living with HIV, there was a cross-cutting consensus in the community health education as something to improve. Another possible focus of study is the improvement of health systems, which is proposed for future research since it was not saturated in this one.

### Community Health Education

Among the participants’ accounts, the perception that *“society does not support you, they discriminate a lot”* was common *(P8, 29 years old, postpartum).* Either by talking badly about PLHIV or by rejecting them.

Asked about what they would like to see changed in order to improve their experience, many expressed a desire for the community to be more educated about HIV.

*I would like society to have more information about this disease and not be afraid” (P4, 31 years old, pregnant)*, [because the participants believe that] “*the community discriminates because they do not understand the advantages of treatment” (P8, 29 years old, postpartum).*

Thus, improving community HIV education was seen as an opportunity to optimize community support for PLHIV.

*I would like to improve the relationship with the community. Because it’s not easy for you to suddenly find out that you have this disease, and you will spend the rest of your life on medication. So, [it would be better] for them to support people more, than to criticize and discriminate. (P7, 20 years old, pregnant).*

The lack of HIV education was also reflected among the negative reactions of the participants in their accounts of the moment they received the diagnosis, with many of them expressing negative feelings such as fear, fright, shock, surprise, and even the desire to take their own lives because they thought it was the end of everything. This is somewhat supported by poor knowledge about HIV and treatment.

*Before [diagnosis], I was looking at the negative side of the disease; I didn’t have any more interactions about it. The negative side I was looking at was how, how is it possible for someone who is HIV- positive to have a child, to get married? Why is that possible? Because I thought it was not possible to control the virus and everything was impossible. I had no information about anything, I was ignorant. (P2, 27 years old, postpartum)*.

Thus, reactions to the diagnosis will depend on the prior information and level of preparation that the participants may have, and negative reactions can be minimized by the support and trust that PLHIV may have from the community, which in turn will depend on the understanding that they may have.

## DISCUSSION

This study investigated the facilitators and barriers faced by women participating in PMTCT programs, in addition to identifying self-expressed possibilities for improvement.

Denial, identified as a barrier, interfered with adherence by delaying treatment initiation in some cases and dropout in others. This finding is similar to that reported in previous studies in Tanzania^(17,18)^, as well as in a study that investigated factors of loss during treatment follow-up in Malawi and found that denial was more common among women without symptoms at the time of diagnosis, leading to the perception that antiretrovirals (ARVs) were not necessary^(19)^.

Although most of the participants reported receiving support from family members, three of the fifteen reported that their spouses did not accept the diagnosis, and as a result they temporarily abandoned ART because they “felt they needed support from them”. This finding was reported in previous studies^(10,19,20)^, emphasizing the importance of support, mainly from partners, as its absence can be a major limiting factor for adherence and retention in HIV care^(8)^.

Women in this study reported side effects as barriers to maintaining treatment, with tolerance depending on the degree of symptoms—feeling more relief than discomfort—and the participants’ health status. This is consistent with studies from Tanzania exploring barriers to treatment adherence^(17–19)^. Poverty, reflected, for example, in the lack of food to meet the necessary demands of ARTs, exacerbated intolerance to side effects. Previous studies in similar settings have also described poverty as a barrier to continue treatment, with many women describing that in order to take ARTs well, they must eat well or else side effects are further intensified^(20,21)^. In the present study, although not reported as a reason for dropout at the time of the study, one woman reported that her daughter had tested positive for HIV because she dropped out of treatment due to lack of food.

Finally, participants identified stigma and discrimination as a challenge they face on a daily basis to remain in the programs and anticipate losses in relation to continuity. This barrier is one of the most reported in many studies in sub-Saharan settings^(8,10,17,19,20,22)^ and persistent over time. Unlike what was observed in one study where it was reported that most participants experienced promulgated stigma^(20,23)^, most participants in the current study experienced anticipated stigma^(23)^. In other studies,^(17)^observed stigma prevailed, where participants witnessed others labeling PLHIV as rotten, devastated, beaten, among other expressions, leading to women disengaging from care for fear of being identified as HIV-positive and discriminated against.

With the intention of finding points that could be strengthened to improve the retention of women in PMTCT programs, factors that facilitate adherence and retention in treatment were identified. Concern for health and family well-being was an important motivator for treatment initiation and follow-up among participants. The finding of this study is consistent with previous studies, conducted in similar settings, which highlight that women, mainly during pregnancy, adhere to treatment out of a desire to protect their babies from HIV infection^(10–12,22,24,25)^. Thus, concern for their health is driven by the desire to care for children and family. That concern for health was reaffirmed by HCPs, as, in most cases, their advice during PMTCT visits focused on the protection and health of the baby, similar to what was reported in the previous study conducted in four different settings in sub-Saharan Africa^(11)^.

Support from families, peers, and HCPs was another motivator reported among participants in this study. Previous work found that support is a factor that enhances persistence or return to treatment in case of previous dropout^(19,22,26)^. Participants in this study were more motivated by treatment when they saw other healthy PLHIV, consistent with that reported in a review of barriers and facilitators to treatment adherence among pregnant women in sub-Saharan Africa^(8)^ which found that peer inspiration was motivating. In addition, having a good relationship with the HCP helps women to better understand their condition, clarify their doubts and overcome the barriers they face at the time of diagnosis, as described by Kiwanuka et al^(22)^.

A novelty of the present study for the Sofala reality is that it also inquired about what the women recommended or wanted to improve in their experiences participating in PMTCTs and what they believed was necessary to support PLHIV. Most of them expressed a desire for the community to become more educated about HIV. Community involvement in the AIDS response is recognized by international AIDS organizations^(27)^ given the impact of this response. Therefore, the level of education the community has, regarding HIV, will largely determine how they perceive and are able to support PLHIV, which can increase support and decrease stigma.

### Strengths and Weaknesses

According to the scientific literature review, this is the first study in the Sofala province to report on the barriers and facilitating factors of pregnant and postpartum women living with HIV and to investigate recommendations for improvement from the perspective of the women themselves. Also, the recruitment methodology that includes women who were attending PMTCT clinics, without being nested in other studies or programs, is novel, as most previous studies followed women who were or had participated in other programs or studies. It is hoped that this recruitment approach has allowed us to give the same possibility of participation to all women enrolled in the programs, independent of their adherence and retention status. Finally, another strength of this study is the researchers’ level of commitment to HIV and their previous research experience in this area.

However, we recognize that, being a virtual-online study where the researchers were not in the same physical location as the participants, it is difficult to state with certainty that there was no interference from third parties—such as the presence of a health care provider—where the interviews were conducted, which could limit the privacy of the participants. However, we are confident that from the confirmation they gave regarding being alone at the time of the interviews, and considering the richness of the accounts provided, it seems that a good level of trust and openness between the parties was achieved, which allowed us to collect reliable data.

## CONCLUSIONS

This study identified that denial (of either diagnosis or treatment), medication side effects, and fear of discrimination continue to limit access, adherence, and retention in care. Participants reported that other people living with HIV also face the same barriers. Future research with other populations may corroborate and complement the findings of this study.

Denial among the participants’ partners was associated with a lack of support. This shows the importance of informing the population so that they can serve as support networks and thus improve adherence among women. Therefore, a relevant strategy that could be implemented in the health services is to carry out health consultations with the pregnant woman’s family in order to educate the whole group and clarify possible doubts.

Finally, factors that facilitated treatment initiation and continuity were identified, such as support from both family members and health care providers. These factors allowed the women to overcome all the barriers they faced and persevere in the programs. Identifying opportunities for improvement from the perception of the women themselves can be an opportunity for change, since these are needs perceived by the affected individuals.

## Financial support

The expenses related to the purchase of equipment for the interviews and compensation of the participants were financed by the School of Nursing’s internal competitive fund, financed by the Postgraduate Department in conjunction with the Research Department, called “Apoyo para Elaboración de Tesis de Magíster en Enfermería (APET)” of the Pontificia Universidad Católica de Chile. Financial support from the Research and Doctorate Directorate of the Nursing School of the Pontificia Universidad Católica de Chile (for publication).
